# Commentary: Early Robotic-Assisted Laparoscopic Pyeloplasty for Infants Under 3 Months With Severe Ureteropelvic Junction Obstruction

**DOI:** 10.3389/fped.2021.724219

**Published:** 2021-08-10

**Authors:** Simone Sforza, Antonio Andrea Grosso, Lorenzo Masieri

**Affiliations:** ^1^Department of Pediatric Urology, Meyer Children Hospital, University of Florence, Florence, Italy; ^2^Unit of Oncologic Minimally-Invasive Urology and Andrology, Department of Experimental and Clinical Medicine, Careggi Hospital, University of Florence, Florence, Italy

**Keywords:** UPJO, pediatric, pyeloplasty, minimally invasive surgery, robot

Dear Prof. Arjan Te Pas,

Dear Dr. Miguel Alfedo Castellan,

We read the recently published article “Early Robotic-Assisted Laparoscopic Pyeloplasty for Infants Under 3 Months With Severe Ureteropelvic Junction Obstruction” with great interest (1). In this study, the authors performed a retrospective study of nine infants under 3 months submitted to robotic-assisted laparoscopic pyeloplasty (RALP) at their institution showing acceptable peri- and postoperative outcomes, including no major complication, a significant decrease in renal pelvis diameter, and improved renal function at 6- and 12-month follow-up.

The use of minimally invasive approach, and in particular the robotic one, to treat benign conditions in the pediatric urology field has tremendously expanded over the last few years with increasing consistent evidence showing comparable successful rate to the open treatment (2, 3) while offering decreased surgical morbidity and better cosmetic result (4). Moreover, as witnessed by several investigations, the indications for robotic correction of ureteropelvic junction obstruction (UPJO) has widened and comprised smaller (<15 kg) and younger (<1 year) infants (5, 6).

In this light, the present study seems to pose a little further cornerstone in the process of expansion of the robotic-assistance in this field. However, some key points need to be clarified. In particular, the authors reported a median operative time (OT) of 109.5 (±10.4) min, a length of hospitalization of 5.57 (±0.73) days, and an overall complication rate of 22% in their series. These outcomes seem to be high as compared with other minimally invasive surgical alternatives, in particular mini-laparoscopic.

Mini-laparoscopic pyeloplasty, indeed, has shown an optimal success rate among several studies, demonstrating a shorter OT and a lower complication rate (7, 8) as compared to the outcomes reported in the present study. Moreover, the mini-laparoscopic approach employs less invasive working ports than the robotic one (3 and 5 vs. 8 mm), translating into better scar acceptance and cosmetic results, which are key outcomes in this specific set of patients and pathologies.

Surprisingly, the authors did not qualitatively assess the cosmetic results in their series, which would have increased the value of the paper.

In addition, other surgical techniques needed to be mentioned since they have been investigated in a similar setting with favorable outcomes and in particular one-trocar-assisted pyeloplasty (OTAP) and open pyeloplasty *via* mini-flank incision (9, 10). Which are the patient-related benefits provided by RALP over these approaches?

On the contrary, as brilliantly discussed by the authors in their paper, RALP has the undoubted advantage of being an easier and reproducible procedure compared to the laparoscopic one, thus leading to a faster learning process; meanwhile, mini-laparoscopic pyeloplasty is burdened by a steep learning curve and thus demanding for highly skilled operators.

To conclude, the management of UPJO in the pediatric urology field is progressively changing, and the latest minimally invasive procedures are replacing the open strategy as the gold standard treatment. However, although RALP has shown enthusiastic results even in the setting of very small and young children, its adoption among infants <3 months has still to be carefully selected since faster surgeries with objectively assessed cosmetic results have been outlined in several papers without compromising the peri- and postoperative outcomes. Further studies with an adequate sample size and, hopefully, in a randomized setting are warranted to draw definitive conclusions.

## Author Contributions

SS, AG, and LM conceived the original idea. SS and AG reviewed the final paper. All authors contributed to the final manuscript.

## Conflict of Interest

The authors declare that the research was conducted in the absence of any commercial or financial relationships that could be construed as a potential conflict of interest.

## Publisher's Note

All claims expressed in this article are solely those of the authors and do not necessarily represent those of their affiliated organizations, or those of the publisher, the editors and the reviewers. Any product that may be evaluated in this article, or claim that may be made by its manufacturer, is not guaranteed or endorsed by the publisher.

## References

[1] Li P Zhou H Cao H Guo T Zhu W Zhao Y. Early robotic-assisted laparoscopic pyeloplasty for infants under 3 months with severe ureteropelvic junction obstruction. Front Pediatr. (2021) 9:590865. 10.3389/fped.2021.590865 33777859 PMC7987794

[2] Masieri L Sforza S Grosso AA Valastro F Tellini R Cini C. Robot-assisted laparoscopic pyeloplasty in children: a systematic review. Minerva Urol Nefrol. (2020) 72:673–90. 10.23736/S0393-2249.20.03854-0 32748621

[3] Dangle PP Kearns J Anderson B Gundeti MS. Outcomes of infants undergoing robot-assisted laparoscopic pyeloplasty compared to open repair. J Urol. (2013) 190:2221–7. 10.1016/j.juro.2013.07.063 23911637

[4] Wang MK Li Y Selekman RE Gaither T Arnhym A Baskin LS. Scar acceptance after pediatric urologic surgery. J Pediatr Urol. (2018) 14:175.e1–6. 10.1016/j.jpurol.2017.11.018 29433993

[5] Masieri L Sforza S Grosso AA Cini C Viola L Tellini R. Does the body weight influence the outcome in children treated with robotic pyeloplasty? J Pediatr Urol. (2020) 16:109.e1–6. 10.1016/j.jpurol.2019.10.023 31806424

[6] Kawal T Srinivasan AK Shrivastava D Chu DI Van Batavia J Weiss D. Pediatric robotic-assisted laparoscopic pyeloplasty: does age matter? J Pediatr Urol. (2018) 14:540.e1–6. 10.1016/j.jpurol.2018.04.023 29909190

[7] Zhou H Liu X Xie H Ma L Zhou X Tao T. Early experience of using transumbilical multi-stab laparoscopic pyeloplasty for infants younger than 3 months. J Pediatr Urol. (2014) 10:854–8. 10.1016/j.jpurol.2013.12.025 24636485

[8] Ludwikowski BM Botländer M González R. The BULT method for pediatric minilaparoscopic pyeloplasty in infants: technique and results. Front Pediatr. (2016) 4:54. 10.3389/fped.2016.00054 27252936 PMC4879137

[9] Lima M Ruggeri G Messina P Tursini S Destro F Mogiatti M. One-trocar-assisted pyeloplasty in children: an 8-year single institution experience. Eur J Pediatr Surg. (2015) 25:262–8. 10.1055/s-0034-1372459 24705997

[10] Bañuelos Marco B Fuller TF Friedersdorff F González R Lingnau A. Transperitoneal mini-laparoscopic pyeloplasty in flank position: a safe method for infants and young adults. Front Surg. (2018) 5:32. 10.3389/fsurg.2018.00032 29725594 PMC5917372

